# Tumours of the Ovary, Fallopian Tube, and Broad Ligament

**Published:** 1884-12

**Authors:** 


*Tumours of the Ovary, Fallopian Tube, and Broad Ligament.* By ALBAN H. G. DORAN, F.R.C.S. Pp. 189. London: SMITH, ELDER & CO. 1884.

Few men are more competent to write a work on ovarian pathology than Mr. Doran. Enjoying unrivalled opportunities at the Samaritan Free Hospital, and making the most of these, he has succeeded in bringing together for examination and study an abundance of material whereon to work out a scientific pathology. His work is ambitious, and justly so. It is evident enough that no one hitherto has aggregated such a mass of facts on his subject as has our author. Not a statement is made nor a conclusion drawn that has not its case or specimen to support it. Everywhere throughout the work there is evidence of careful observation and honest labour in the recording of minutiæ, and thus far it has no rival in the subject on which it treats.

Saying so much, and admitting heartily that it is the best book we have on the subject, we are still disappointed that it is not better. The fault we find with it is that there are too many minutiæ, too many isolated facts, and not enough generalisation. All through the work we are noting individual specimens, reading of individual cases; but rarely do we meet with conclusions built on these units, or when we do they are expressed in such meagre or disjointed terms that they neither impress us nor carry conviction. And there is a want of clear arrangement and method in the first parts of the work, which are most harassing to the reader. The sentences are clear enough; but the paragraphs are confused, and the chapters are simply muddled.

It grieves us to have to write thus of a work of such surpassing excellence generally, and we do so in the hope that a future edition may show an absence of these defects. We could spare the chapters relating to operation, and would be glad to see their space occupied by some philosophical statements of broad pathology such as, we are sure, Mr. Doran could easily produce. Perhaps, when a year or two have passed by, the individual facts will appear less overwhelming, and their generalised bearings will become more prominent.

We reiterate our opinion, that, in spite of its defects, this is the best work on ovarian pathology in existence. We have learnt more from its perusal than we have done from all previous writings on the subject; our only disappointment is, that with his evident capacity to do so, the author has not taught us more.

